# Cardiac Tamponade in Late Pregnancy Caused by *Corynebacterium amycolatum* Pericarditis and Managed by a Surgical Pleuro-Pericardial Window

**DOI:** 10.3390/jcm15145407

**Published:** 2026-07-10

**Authors:** Adam Ryszard Kowalówka, Tomasz Gallina, Anna Kaźmierska, Aleksandra Michalewska-Włudarczyk, Maciej Kaźmierski, Wojciech Wojakowski, Radosław Gocoł

**Affiliations:** 1 Department of Cardiac Surgery, Upper-Silesian Medical Centre, Medical University of Silesia, Ziołowa 45-47, 40-635 Katowice, Poland; gocot@poczta.onet.pl; 2 3rd Department of Cardiology, Upper-Silesian Medical Centre, Medical University of Silesia, Ziołowa 45-47, 40-635 Katowice, Poland; tomasz.gallina@gmail.com (T.G.); ania.kazmierska43@gmail.com (A.K.); amichalewska1@gmail.com (A.M.-W.); kazmierski.maciej@gmail.com (M.K.); wojtek.wojakowski@gmail.com (W.W.)

**Keywords:** cardiac tamponade, pregnancy, *Corynebacterium amycolatum*, pericarditis, pericardial window, obesity, echocardiography, heart team

## Abstract

Cardiac tamponade in pregnancy is an exceptional maternal–fetal emergency in which physiological tachycardia, hypervolaemia and dependent oedema mask the classical signs of tamponade. *Corynebacterium amycolatum* is a non-diphtherial, Gram-positive coryneform commensal of human skin and mucosa that is increasingly recognised as a true invasive pathogen, although pericardial infection has rarely, if ever, been reported. We aimed to describe the diagnostic and decompression strategy in such a case. We report a 29-year-old woman at 30 + 4 weeks of gestation with class III obesity (pre-pregnancy body mass index 36 kg/m^2^), pregnancy-induced hypertension and diet-controlled type 2 diabetes referred after a routine echocardiogram suggested tamponade despite preserved haemodynamic compensation. Transthoracic echocardiography demonstrated a large circumferential pericardial effusion with diastolic right-atrial and right-ventricular collapse, a plethoric inferior vena cava and respiratory mitral-inflow variation. Severe maternal obesity superimposed on advanced gestation degraded the acoustic windows, elevated the diaphragm, displaced the heart anteriorly and brought the gravid uterus into the subxiphoid corridor, rendering percutaneous pericardiocentesis prohibitively hazardous. After multidisciplinary heart-team review, a left anterior mini-thoracotomy with pleuro-pericardial window evacuated approximately 1000 mL of fluid. Aerobic culture yielded *C. amycolatum* (MALDI-TOF), with histological pericarditis and a consistent antibiogram; autoimmune, viral and neoplastic causes were excluded, although blood cultures and extended viral testing were not performed. Targeted intravenous cefazolin was given, and the patient delivered a healthy term neonate at 39 weeks, with a normal 7-month echocardiogram. To the best of our knowledge, this is among the first reported cases of *C. amycolatum* pericardial tamponade in pregnancy. Because blood cultures and molecular confirmation of pericardial involvement were not obtained, a contaminant or incidental role for the organism cannot be entirely excluded, and the causal attribution should be regarded as probable rather than definitive. The case highlights heart-team-based individualised decompression and the cautious microbiological interpretation of organisms traditionally regarded as commensals.

## 1. Introduction

Small, physiological pericardial effusions of 1–2 mm—occasionally up to 10 mm—are detected echocardiographically in up to approximately 40% of women during the third trimester and are generally of no clinical consequence [1]. By contrast, pericardial tamponade in pregnancy is exceedingly rare and constitutes a maternal–fetal emergency. Pregnancy-related symptoms like: physiological adaptation, tachycardia, expanded plasma volume and dependent oedema frequently obscure the classical clinical signs of tamponade, so that a haemodynamically significant effusion may be reached while the patient remains apparently well [2]. The aetiological spectrum in pregnancy mirrors that of the general population (malignancy, viral and bacterial pericarditis, autoimmune disease and iatrogenic injury), with an additional contribution from pregnancy-specific hypertensive disorders such as pre-eclampsia and HELLP syndrome [3].

*Corynebacterium amycolatum* is a non-diphtherial, Gram-positive coryneform bacillus that forms part of the normal cutaneous and mucosal flora and is frequently dismissed as a culture contaminant [4,5]. Over the past two decades, however, it has been increasingly established as a genuine invasive pathogen, causing bacteraemia, prosthetic-device infection, infective endocarditis and, most recently, early-onset neonatal sepsis [6,7,8]. Despite this expanding clinical footprint, *C. amycolatum* has—to the best of our knowledge—rarely, if ever, been reported as a cause of pericarditis or pericardial tamponade, and we are not aware of any previous report in the context of pregnancy [9,10,11]. Following a focused search of PubMed/MEDLINE (search terms *“Corynebacterium amycolatum”* combined with “pericarditis”, “pericardial effusion”, “tamponade” and “pregnancy”; English-language literature; last searched June 2026), we identified no prior description of this organism as a pericardial pathogen. We therefore report what is, to the best of our knowledge, among the first cases of pericardial tamponade in pregnancy attributable to *Corynebacterium amycolatum*, in which severe maternal obesity compounded by advanced gestation precluded percutaneous drainage and a surgical pleuro-pericardial window was performed instead. The case therefore unites two separate rarities—an unusual pericardial pathogen and a high-risk decompression dilemma—in a single pregnant patient.

## 2. Case Presentation

A 29-year-old gravida 2, para 1 woman at 30 + 4 weeks of gestation was referred to the Emergency Department of the Upper-Silesian Medical Centre, Medical University of Silesia, Katowice, Poland, after a routine outpatient echocardiogram suggested cardiac tamponade. She had been followed in the cardiology clinic for pregnancy-induced hypertension. Her history was notable for class III obesity (pre-pregnancy body mass index 36 kg/m^2^; gestational weight gain approximately 8 kg), type 2 diabetes mellitus diagnosed three years earlier and currently diet-controlled, and an unspecified febrile infection approximately one month before admission. Her medications were 250 mg methyldopa three times daily and 75 mg acetylsalicylic acid once daily. Since early pregnancy she had noticed intermittent palpitations and progressive exertional dyspnoea, both attributed at the time to physiological gestational changes.

**Figure 1:**
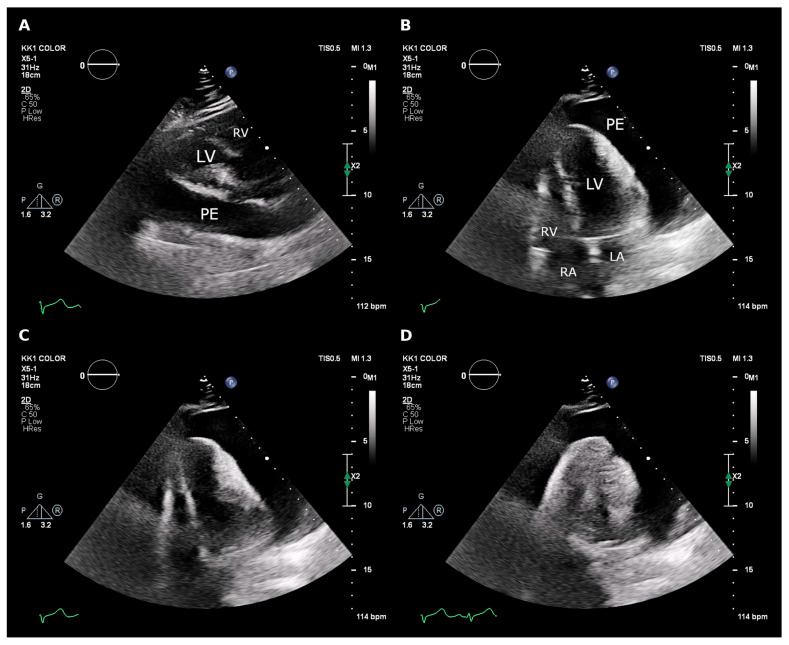
Preoperative transthoracic echocardiographic features of cardiac tamponade in a 29-year-old woman at 30 + 4 weeks of gestation (all panels obtained at presentation, before surgical drainage). (**A**) Parasternal long-axis view demonstrating a large pericardial effusion (PE) measuring up to 30 mm posterior to the left ventricle. (**B**–**D**) Sequential apical four-chamber frames showing a circumferential pericardial effusion of up to 40 mm around the apex and lateral wall, with diastolic collapse of the right atrium and right ventricle. LA, left atrium; LV, left ventricle; PE, pericardial effusion; RA, right atrium; RV, right ventricle.

**Figure 2:**
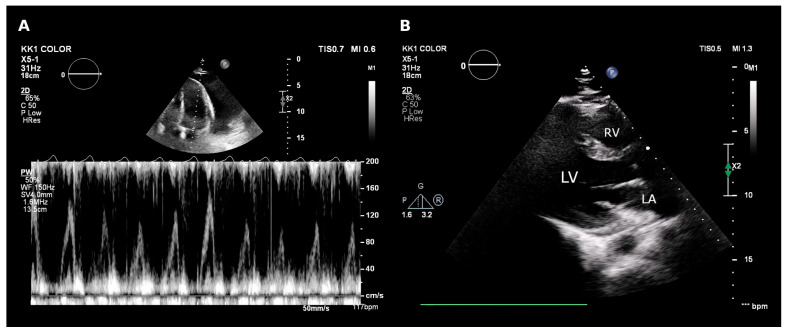
Doppler confirmation of tamponade physiology and postoperative resolution of the effusion. (**A**) Pulsed-wave Doppler tracing of mitral inflow at presentation (30 + 4 weeks of gestation) showing marked respiratory variation in peak E-wave velocity, consistent with tamponade physiology. (**B**) Parasternal long-axis view on the first postoperative day, after left anterior mini-thoracotomy with pleuro-pericardial window, showing resolution of the pericardial effusion. LA, left atrium; LV, left ventricle; RV, right ventricle.

On arrival the patient was alert, fully oriented and in no respiratory distress. Blood pressure was 150/80 mmHg, heart rate 110 bpm in sinus rhythm, peripheral oxygen saturation 96% on room air and tympanic temperature 36.9 °C. Cardiac auscultation revealed muffled heart sounds without pathological murmurs; vesicular breath sounds were symmetrical. Bilateral lower-limb pitting oedema was present. The uterine fundus was palpable three fingerbreadths above the umbilicus and the fetal heart rate was regular at 130 bpm. The 12-lead electrocardiogram showed sinus rhythm at 105 bpm with low-amplitude R waves across all precordial leads (R-wave amplitude 2 mm in V3 and 6 mm in V5-V6) and a negative T wave in V1, consistent with the low-voltage pattern of a large pericardial effusion. Transthoracic echocardiography in the emergency department, confirmed in the intensive care unit using the focused transthoracic echocardiography in emergency (FATE) protocol, demonstrated a large circumferential pericardial effusion measuring up to 30 mm posterior to the left ventricle, 16 mm anterior to the right ventricle and up to 40 mm around the apex and lateral wall, with diastolic collapse of the right atrium and right ventricle and a plethoric, non-collapsing inferior vena cava (Figure 1A–D). Pulsed-wave Doppler interrogation of mitral inflow showed marked respiratory variation in peak E-wave velocity, confirming tamponade physiology (Figure 2A). Left ventricular ejection fraction was preserved at approximately 50%. Crucially, the same study quantified procedural risk: deep subcutaneous and prepericardial soft tissue attenuated the ultrasound beam and degraded the subxiphoid and apical windows, gestational diaphragmatic elevation displaced the heart anteriorly, and the effusion was loculated with a predominantly posterior distribution.

Laboratory investigations showed normocytic anaemia (haemoglobin 9.5 g/dL, haematocrit 29.3%), mild hypokalaemia (potassium 3.2 mmol/L), modestly elevated inflammatory markers (C-reactive protein 17.2 mg/L, interleukin-6 3.9 pg/mL, procalcitonin 0.08 ng/mL) and a mildly elevated high-sensitivity troponin T (0.035 ng/mL). The white-cell count, platelet count, creatinine (0.61 mg/dL), sodium (138 mmol/L), NT-proBNP, thyroid function, hepatic enzymes, coagulation parameters (INR 1.1, activated partial thromboplastin time 28.1 s), lactate (1.4 mmol/L) and urinalysis—including assessment for proteinuria—were within the reference range. Combined nasopharyngeal swabs for SARS-CoV-2 and influenza A/B were negative.

In view of the recent febrile illness and the pregnancy, the differential diagnosis encompassed: viral or bacterial pericarditis; systemic autoimmune disease, particularly systemic lupus erythematosus, the best-documented cause of pericardial tamponade in pregnancy [4]; malignant pericardial involvement, such as occult metastatic disease; pregnancy-specific hypertensive complications including preeclampsia and HELLP syndrome [10,11]; and iatrogenic or traumatic causes. Aortic dissection and uraemic pericarditis were considered unlikely on clinical and biochemical grounds. Rare inflammatory and histiocytic disorders—most notably Erdheim–Chester disease and related non-Langerhans-cell histiocytoses—are recognised but underdiagnosed causes of pericardial infiltration and constrictive pericarditis that can mimic chronic infectious pericardial disease, particularly when pleural involvement coexists, and were therefore also considered in the differential of this patient with combined with pleuro-pericardial disease [12]. The specific investigations used to address each diagnostic hypothesis are summarised in Table 1.

The patient was admitted to the cardiothoracic intensive care unit for invasive arterial pressure monitoring, continuous telemetry and strict fluid balance. Cautious crystalloid resuscitation was administered to optimise preload while avoiding volume overload, and potassium was replaced. Blood pressure was controlled with an intravenous urapidil infusion (10 mg/h; target systolic 130–150 mmHg), and intravenous magnesium sulphate was started per the obstetric preeclampsia prophylaxis protocol (4 g loading dose followed by 1 g/h). Right internal jugular central venous access was obtained, and continuous external cardiotocography confirmed reassuring fetal status throughout.

An emergency multidisciplinary heart-team consultation was convened, comprising a cardiologist, a cardiac anaesthetist, a cardiac surgeon, an obstetrician and a neonatologist. Although the patient remained haemodynamically stable, repeat echocardiography between hours 8 and 13 demonstrated persistent tamponade physiology. The team explicitly considered percutaneous pericardiocentesis as the standard first-line intervention but judged it to carry a prohibitive risk profile in this individual patient. First, class III obesity (body mass index 36 kg/m^2^) produced a markedly thick subcutaneous and pre pericardial soft tissue layer that severely limited the subxiphoid and apical acoustic windows, precluding real-time, high-resolution ultrasound guidance of the needle tip. Second, advanced gestation (30 + 4 weeks) had elevated and rotated the diaphragm, displaced the heart anteriorly and to the left, and brought the gravid uterus into close proximity to the subxiphoid puncture site, lengthening the needle trajectory and increasing the risk of myocardial, coronary, hepatic and of particular concern inadvertent uterine and fetal injury. Third, the loculated, predominantly posterior distribution of the effusion further reduced the safety margin of a blind or partially imaged percutaneous approach. After balancing these procedural risks against the patient’s preserved haemodynamic compensation, the heart team elected to proceed directly to surgical drainage. The team recognised that general anaesthesia and surgical decompression carry their own fetal risks—chiefly maternal hypotension with reduced utero-placental perfusion and the possibility of provoking preterm labour—and these were explicitly weighed against the procedural hazards of percutaneous drainage. A fetal-protection anaesthetic strategy was therefore adopted: left-uterine displacement to avoid aortocaval compression, maintenance of maternal normotension and normocapnia with vigilant avoidance of hypotension, pre-oxygenation and rapid-sequence induction, use of agents with an established gestational-safety profile, continuous intra-operative cardiotocographic monitoring of fetal heart rate, and immediate availability of obstetric and neonatal teams for emergency caesarean delivery should fetal compromise occur. Magnesium sulphate already in progress for pre-eclampsia prophylaxis additionally provided a degree of tocolytic cover. Under general anaesthesia, with full obstetric and neonatal stand-by, a left anterior mini-thoracotomy was performed in the fourth intercostal space and a pleuro-pericardial window was created. Approximately 1000 mL of straw-coloured pericardial fluid was evacuated, with samples sent for microbiology, cytology and histopathology; the pericardium was biopsied and a single pleural drain was left in situ. The patient was extubated in theatre and transferred to the intensive care unit in a stable condition.

The early postoperative course was uneventful. On the first postoperative day the patient reported wound pain (5/10 on the numeric rating scale) but no dyspnoea or chest pain, and remained haemodynamically stable on 10 mg/h intravenous urapidil and 25 mg oral metoprolol succinate every 8 h. Cardiotocography demonstrated a reactive, normal fetal heart-rate pattern. Follow-up echocardiography on postoperative day 1 showed only a small residual pericardial effusion (≤4 mm anterior to the right ventricle), preserved left ventricular ejection fraction (approximately 50%), a small left pleural effusion and minor basal atelectasis; the pleural drain was removed once drainage became minimal (Figure 2B). Postoperative laboratory values were unremarkable, with a normal white-cell count (10.12 × 10^9^/L) and a stable haemoglobin count.

Pericardial fluid obtained intra-operatively under sterile conditions was sent directly for microbiological processing. Aerobic culture grew *Corynebacterium amycolatum*; anaerobic cultures were negative after 5 days. The organism was identified by matrix-assisted laser desorption/ionisation-time-of-flight mass spectrometry (MALDI-TOF). Antimicrobial susceptibility testing, interpreted according to the prevailing EUCAST breakpoints, demonstrated sensitivity to penicillin, ciprofloxacin, tetracycline and linezolid, with resistance to clindamycin. Histopathology of the pericardial specimen revealed thickening of the fibrous layer, vascular congestion with marginated leukocytes and transmural migration, and small foci of haemorrhage—consistent with non-specific pericarditis and without evidence of malignancy. An extended autoimmune and infectious work-up—antinuclear antibodies (HEp-2), anti-dsDNA, p-ANCA, c-ANCA, anti-CCP and anticardiolipin antibodies, and serology for HIV, hepatitis B and C, *Treponema pallidum* and *Toxoplasma gondii*—was uniformly negative. The patient was discharged from cardiac surgery on the sixth postoperative day and transferred to obstetrics, where targeted intravenous cefazolin (1 g every 8 h) was initiated in line with the antibiogram and pregnancy-safe antimicrobial principles. Cefazolin was preferred over alternative agents to which the isolate was susceptible because, in addition to the favourable antibiogram, it combines a well-established category-B gestational-safety profile, reliable activity against Gram-positive organisms and good penetration into serous spaces, whereas tetracycline is contraindicated in pregnancy and fluoroquinolones and linezolid are generally avoided. A total antimicrobial course of approximately four weeks (six days intravenously in hospital followed by completion as guided by the obstetric and infectious-disease teams) was planned for culture-proven purulent pericarditis. Therapy was well tolerated and the patient was discharged home after a further 10 days of obstetric observation.

At the 30-day cardiology review the patient was at 34 + 4 weeks of gestation, free of cardiovascular and respiratory symptoms, with a well-healed wound and a normal echocardiogram (left ventricular ejection fraction 60%, no residual pericardial or pleural effusion). The remainder of the pregnancy was uncomplicated, and she delivered a healthy term neonate by spontaneous vaginal delivery at 39 weeks. At the 7-month cardiology follow-up the echocardiogram remained normal, with no recurrence of pericardial effusion and preserved systolic function. Given the purulent aetiology and the recognised risk of constrictive pericarditis after bacterial pericarditis, a structured surveillance strategy was planned, comprising serial transthoracic echocardiography with assessment for constrictive physiology (respiratory septal shift, annulus reversus, dilated non-collapsing inferior vena cava) and clinical review, with cross-sectional imaging (cardiac magnetic resonance or computed tomography) reserved for any symptoms or echocardiographic features suggestive of evolving constriction. No clinical or echocardiographic evidence of constriction had emerged by the 7-month follow-up.

This report unites three uncommon strands: an exceptional obstetric emergency, a high-risk decompression dilemma, and an unusual pericardial pathogen within a single patient. Although small, physiological pericardial effusions are detected in up to 40% of third-trimester pregnancies; progression to haemodynamically significant tamponade is exceptional [1]. The clinical impact of an effusion depends less on its absolute volume than on the rate of accumulation and the biomechanical compliance of the pericardium: rapidly accumulating effusions cause a steep early rise in intra-pericardial pressure, whereas chronic effusions may reach considerable volumes with few symptoms because of adaptive pericardial stretching [3].

The dominant aetiologies of pericardial tamponade in the general population—malignancy, infectious pericarditis, iatrogenic complications and autoimmune disease, especially systemic lupus erythematosus—are also encountered in pregnancy, with an additional contribution from pregnancy-specific hypertensive disorders [3,10,11]. Wichert-Schmitt and colleagues reported a 37-year-old woman at 24 weeks in whom thoracoscopic pericardial fenestration unmasked previously undiagnosed lupus [4]; Romagano and colleagues described a previously healthy woman who developed tamponade at 32 weeks, managed by a multidisciplinary team with a surgical pericardial window and drainage [9]. In our patient, autoimmune, viral and neoplastic aetiologies were systematically excluded by negative serological, cytological and histopathological investigations, whereas pericardial fluid culture identified *Corynebacterium amycolatum* as the most likely causative organism. The exclusion of neoplastic involvement merits particular emphasis, because pericardial masses and pseudotumoral lesions can closely mimic infectious or malignant pericardial disease and represent an important differential in complex pericardial presentations. In the broader assessment of suspected cardiac and pericardial masses, an integrated multimodality imaging approach—combining echocardiography, cardiac magnetic resonance, cardiac computed tomography and ^18^F-fluorodeoxyglucose positron emission tomography—improves diagnostic accuracy, with cardiac magnetic resonance offering the highest standalone accuracy for malignancy and positron emission tomography adding value in inconclusive cases [13]. In the present patient, the absence of any discrete mass, infiltrative process or pathological tissue on echocardiography and contrast-enhanced computed tomography, together with negative pericardial fluid cytology and benign pericardial histopathology, was considered sufficient to confidently exclude neoplastic pericardial involvement; a more structured multimodality work-up (notably cardiac magnetic resonance or ^18^F-fluorodeoxyglucose positron emission tomography) would have been triggered had the imaging or histological findings been equivocal. Such escalation is, however, particularly delicate in pregnancy. Although malignancy in pregnancy remains rare, its incidence is rising, and the diagnostic and therapeutic pathway is constrained by the need to limit fetal exposure to ionising radiation and to weigh the fetal risks of staging investigations and treatment against the maternal benefit, so that imaging modalities free of ionising radiation (echocardiography and, where required, non-contrast cardiac magnetic resonance) are preferred and invasive or radiation-based work-up is reserved for cases in which the clinical suspicion of malignancy is high [14]. In our patient, the convergent non-neoplastic findings allowed this dilemma to be resolved without recourse to fetus-hazardous investigations.

A defining feature of this case was the marked dissociation between echocardiographic severity and clinical presentation. Despite clear echocardiographic evidence of tamponade physiology, the patient remained haemodynamically compensated, with no chest pain and only mild dyspnoea and palpitations—both readily attributable to pregnancy. The physiological tachycardia, hypervolaemia and dependent oedema of pregnancy mask the classical Beck triad and pulsus paradoxus, so a high index of suspicion is mandatory: even non-specific, late-pregnancy symptoms warrant prompt echocardiography when findings such as muffled heart sounds, a low-voltage QRS or unexplained tachycardia raise the possibility of pericardial pathology [3]. In this regard, the readily available surface 12-lead electrocardiogram deserves particular emphasis, as it can shorten the time to diagnosis when echocardiography is not immediately to hand. During tamponade the electrocardiogram may demonstrate low QRS voltage and electrical alternans—the beat-to-beat variation in QRS amplitude produced by the pendular swinging of the heart within a large effusion [15]. Sinus tachycardia is almost universal, and arrhythmias including premature ventricular complexes and supraventricular tachycardia may also be observed, reflecting mechanical irritation and haemodynamic stress on the myocardium [16]. In our patient the electrocardiogram indeed showed sinus tachycardia and a low-voltage pattern across the precordial leads, findings that—if actively sought—can corroborate clinical suspicion and prompt expedited echocardiographic confirmation, even though they are neither sensitive nor specific in isolation.

The microbiological finding is the principal novelty of this report. *Corynebacterium amycolatum* is a non-diphtherial, Gram-positive coryneform bacillus of normal cutaneous and mucosal flora [5]. Although traditionally dismissed as a contaminant, it is now firmly recognised as a true pathogen in invasive infections such as bacteraemia, prosthetic-device infection, notably the first reported case of corynebacterial endocarditis caused by this species, and, most recently, early-onset neonatal sepsis [6,7,8]. Multidrug resistance is characteristic and complicates therapy. In our case the organism was recovered from pericardial fluid obtained intra-operatively under sterile conditions, identified by MALDI-TOF, and supported by histological pericarditis and an internally consistent antibiogram; taken together these findings argue against simple skin contamination and in favour of genuine pericardial infection, although a contaminant or incidental role cannot be excluded with certainty in the absence of blood cultures or molecular confirmation. Pregnancy-related modulation of cell-mediated immunity, together with an increased propensity for fluid accumulation in serous spaces, may have facilitated clinical infection by an otherwise-low-virulence commensal. A focused review of the literature identified reports of *C. amycolatum* in endocarditis, bacteraemia, peritonitis, device-related infection and neonatal sepsis, but none describing pericarditis or pericardial tamponade, and none in pregnancy [5,6,7,8]. To the best of our knowledge, the present case is therefore among the first documented instances of *C. amycolatum* associated with pericardial tamponade in pregnancy.

The cornerstone of treatment of cardiac tamponade is urgent decompression of the pericardial space, and percutaneous pericardiocentesis is the recommended first-line intervention [3]. In this patient, three converging factors rendered it prohibitively hazardous and prompted the heart team to proceed directly to surgical drainage. The combination of severe maternal obesity and advanced gestation produced an exceptionally challenging procedural environment: the subxiphoid and apical acoustic windows were formally assessed during the diagnostic echocardiogram and judged inadequate for safe, image-guided needle placement, as the thick chest wall and adipose layer attenuated the ultrasound beam and limited real-time needle visualisation; diaphragmatic elevation and anterior cardiac displacement shortened the safe corridor between the parietal pericardium and vital structures; and the gravid uterus encroached on the subxiphoid window, lengthening the needle trajectory and exposing the fetus to direct mechanical injury in the event of misdirection. The predominantly posterior distribution of the effusion further reduced the technical safety margin, and the analgesia/sedation required for a difficult percutaneous procedure carried a non-negligible risk to fetal perfusion. A surgical pleuro-pericardial window through a left anterior mini-thoracotomy provided definitive decompression under direct vision, allowed comprehensive fluid and tissue sampling for microbiology, cytology and histopathology, and minimised the risk of recurrent effusion—an approach consistent with individualised, heart-team-based decision-making for cardiovascular disease in pregnancy [3,11]. Once a bacterial aetiology was confirmed, targeted intravenous cefazolin—an antibiotic with an established pregnancy-safety profile and demonstrated activity against the isolate—was initiated in agreement with the antibiogram and continued as a definitive course in conjunction with the obstetric and infectious-disease teams.

## 3. Limitations

As a single-patient case report, the generalisability of these observations is inherently limited. The principal methodological limitation concerns the microbiological attribution: blood cultures were not obtained, and molecular confirmation (for example, broad-range 16S rRNA gene sequencing) of pericardial involvement was not performed. Consequently, although the organism was recovered from pericardial fluid sampled intra-operatively under sterile conditions and was supported by histological pericarditis and an internally consistent antibiogram, a contaminant or incidental role for *Corynebacterium amycolatum* cannot be formally excluded, and the causal inference should therefore be regarded as probable rather than definitive. An extended viral panel beyond the SARS-CoV-2 and influenza swabs and serologies obtained was likewise not undertaken.

## 4. Conclusions

We describe what is, to the best of our knowledge, among the first reported cases of cardiac tamponade in pregnancy attributable to *Corynebacterium amycolatum*, successfully managed by a left anterior mini-thoracotomy with pleuro-pericardial window in a patient in whom severe maternal obesity combined with advanced gestation made percutaneous pericardiocentesis unsafe. The case underscores the importance of a high index of suspicion for pericardial pathology when late-pregnancy symptoms are non-specific; heart-team-based, individualised choice of decompression strategy that explicitly weighs maternal habitus, gestational age and effusion characteristics; and careful microbiological interpretation of organisms traditionally regarded as commensals when supported by appropriate clinical, sterilely obtained microbiological and histopathological evidence.

## Figures and Tables

**Table 1:** Summary of the differential diagnosis and the investigations used to exclude each aetiology of pericardial tamponade in this patient.

Differential Diagnosis	Investigations Performed	Result
Bacterial pericarditis	Aerobic/anaerobic culture and MALDI-TOF of intra-operative pericardial fluid; histopathology; inflammatory markers (CRP, IL-6, PCT)	Confirmed: *C. amycolatum* on culture with consistent antibiogram and histological pericarditis
Viral pericarditis	Nasopharyngeal swabs for SARS-CoV-2 and influenza A/B; serology for HIV and hepatitis B and C	Excluded (negative); extended viral panel not performed
Systemic autoimmune disease (e.g., SLE)	ANA (HEp-2), anti-dsDNA, p-ANCA, c-ANCA, anti-CCP, anticardiolipin antibodies	Excluded (uniformly negative)
Malignant/neoplastic involvement	Pericardial fluid cytology; histopathology of pericardial biopsy; echocardiographic and CT-based morphological assessment	Excluded (no malignant cells; no mass on imaging)
Histiocytic disease (e.g., Erdheim–Chester)	Pericardial histopathology; absence of infiltrative/constrictive imaging features	Considered unlikely (no histiocytic infiltrate or constriction)
Pregnancy-specific hypertensive disorders (pre-eclampsia/HELLP)	Blood pressure, urinalysis/proteinuria, platelet count, hepatic enzymes, haemolysis markers	Excluded (no proteinuria, normal platelets and liver enzymes)
Aortic dissection/uraemic/iatrogenic–traumatic	Clinical assessment, echocardiography/CT, renal function (creatinine), absence of recent instrumentation or trauma	Considered unlikely on clinical and biochemical grounds

ANA, antinuclear antibodies; anti-CCP, anti-cyclic citrullinated peptide; anti-dsDNA, anti-double-stranded DNA; c-ANCA/p-ANCA, cytoplasmic/perinuclear anti-neutrophil cytoplasmic antibodies; CRP, C-reactive protein; CT, computed tomography; HELLP, haemolysis, elevated liver enzymes and low platelets; IL-6, interleukin-6; MALDI-TOF, matrix-assisted laser desorption/ionisation–time-of-flight mass spectrometry; PCT, procalcitonin; SLE, systemic lupus erythematosus.

## Data Availability

All data supporting the findings of this case report are contained within the article. Further enquiries can be directed to the corresponding author.
